# Stevens–Johnson Syndrome and Herpes Simplex Type 1 Infection during Adalimumab Therapy for Crohn's Disease

**DOI:** 10.1155/2020/3875024

**Published:** 2020-04-15

**Authors:** Jenny Roselli, Tommaso Innocenti, Erica Nicola Lynch, Laura Parisio, Pasquale Apolito, Tommaso Mello, Giuseppe Macrì, Monica Milla, Maria Rosa Biagini, Mirko Tarocchi, Stefano Milani, Andrea Galli

**Affiliations:** ^1^Department of Biomedical, Experimental and Clinical Sciences, University of Florence, Florence, Italy; ^2^Azienda Ospedaliero-Universitaria Careggi, Florence, Italy

## Abstract

Stevens–Johnson syndrome (SJS) is a severe mucocutaneous adverse drug reaction with a relatively high mortality rate. SJS is described during herpes simplex virus type 1 (HSV1) infection and, rarely, even during adalimumab therapy. We report the case of a patient with Crohn's disease who developed SJS during an HSV1 infection and a contemporaneous anti-TNF*α* therapy with adalimumab. Remission was achieved with suspension of adalimumab and high doses of intravenous steroids and antivirals. Patients with HSV1 infection and on adalimumab therapy have a combined risk of SJS and should be monitored closely.

## 1. Introduction

Stevens–Johnson syndrome (SJS) is a severe mucocutaneous adverse reaction, characterized by necrosis of skin and mucosa upper layers, with consequent formation of vesicles, bullae, and ulcers. In SJS, skin detachment involves less than 10% of the body surface area [1]. Common initial clinical manifestations of SJS are malaise, fatigue, persistent high fever, skin tenderness, and pain. In SJS, the primary immunological barrier of the skin is disrupted and there is an increased risk of infections [2, 3]. SJS is considered a severe drug reaction, as drugs are its most common trigger [4, 5]. It is a medical condition that usually requires hospitalization and that has a mortality rate of 1–5% [6].

We describe the case of an IBD patient who developed SJS during a herpes simplex type 1 infection and a contemporary anti-TNF alpha therapy with adalimumab.

## 2. Case Report

**Figure 1:**
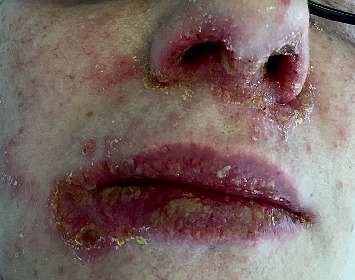
Perioral desquamative lesions.

**Figure 2:**
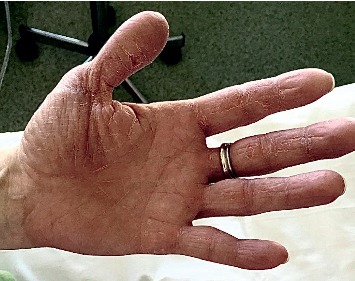
Palmar desquamative lesions.

A 44-year-old female patient was diagnosed with ileocolonic steno-penetrating Crohn's disease in 2006. She also suffered from dermatological manifestations of the disease (erythema nodosum and psoriasiform dermatitis). Initially, the patient was treated with systemic steroids and mesalamine and achieved a good clinical response. Subsequently, mesalamine was interrupted due to drug intolerance. In 2007, the disease flared up, so the patient underwent an ileocolonic resection. No postoperative treatment was initiated. The patient was first referred to our Inflammatory Bowel Disease Center (AOU Careggi, Florence) in 2015 because of a disease relapse. In order to assess disease activity, a colonoscopy, abdomen CT scan, and digestive MRI scan were carried out, which revealed a 8–10-cm long preanastomotic stricture. Thus, in October 2015, the patient started treatment with adalimumab. After three months of therapy, the patient was admitted to our hospital with malaise, high fever, pain, pruritus, and skin and mucosa detachment of <5% of body surface area (mouth, periorbital region, palms, and soles) with positive Nikolsky sign; (Figures 1 and 2) she also presented with vesicular lesions of the nostrils and nasolabial fold. The patient underwent a vesicle swab, which demonstrated the presence of HSV1 DNA. The patient was evaluated by our consultant Dermatologist and was diagnosed with herpes simplex type 1 infection complicated by bacterial superinfection with impetigo and SJS. Isolation precautions were used because of the patient's high risk of acquiring infection. The patient was treated with fluids, antivirals, and high doses of intravenous steroids until she achieved complete remission. This event could either have been a consequence of the herpes simplex type 1 infection, or an adverse reaction to adalimumab, therefore, in this eventuality, the treatment was discontinued. Nevertheless, we decided to start treatment with infliximab in December 2017, after consulting our Immunologist who confirmed the absence of contraindications to the treatment with a different anti-TNF alpha drug. She is currently still being treated with infliximab and regularly attends follow-up visits at our Outpatient Department. She has not developed any adverse events to the drug.

## 3. Discussion

Skin reactions to adalimumab are frequent and extremely varied. The most common dermatologic adverse event to adalimumab is injection-site erythema and pruritus, which resolve spontaneously and occur less frequently with time. Conversely, eczema and psoriasiform lesions can be so severe as to cause treatment interruption [7].

In the literature, only two other cases of SJS during adalimumab therapy are described: Mounach et al. presented the case of a 53-year-old patient who was treated with adalimumab for rheumatoid arthritis [8], while Salama and Lawrance described the case of a 29-year-old patient with ileocolonic steno-penetrating Crohn's disease, with dermatological and rheumatological extraintestinal manifestations [9].

In both these cases, the clinical manifestations were pathognomonic, with peripheral rash, abdominal skin detachment, and oral cavity mucositis, so that biopsy was not necessary for diagnosis. In both these cases, as well as in our, SJS resolved with the suspension of adalimumab and with the administration of high doses of intravenous steroids; the patients were then switched to infliximab therapy without complications [8, 9].

On the other hand, herpes simplex type 1 infection is also a well-known cause of SJS and one of the most frequent triggers of erythema multiforme [10, 11]. In 1992, Detjen et al. described a case of SJS in a 36-year-old man with recurrent HSV-related oropharyngitis; even in this case, as in ours, the patient was treated with antivirals and steroids, achieving complete resolution of signs and symptoms [12].

## 4. Conclusions

SJS is a severe medical condition, described during HSV type 1 infection and, rarely, even during adalimumab therapy.

Patients in treatment with biologic drugs, such as adalimumab, are more susceptible to recurrent reactivation of HSV type 1 infection. As a result, they have a combined risk of SJS. Patients who are treated with adalimumab should be monitored closely, especially in case of simultaneous therapy with other drugs, and should be advised to seek immediate medical care if alarming symptoms appear.
